# Association between health literacy and medication comprehension; attitudes toward reporting adverse events in adults using over-the-counter medicines

**DOI:** 10.1186/s40545-023-00596-3

**Published:** 2023-07-17

**Authors:** Shoichi Masumoto, Tomotsugu Yamakawa, Naoto Sakamoto, Tetsuhiro Maeno

**Affiliations:** 1 Department of Family Medicine, General Practice and Community Health, Institute of Medicine, Faculty of Medicine, University of Tsukuba, 1-1-1 Tennodai, Tsukuba, Ibaraki 305-8575 Japan; 2 Department of General Medicine, Tsukuba Central Hospital, 1589-3 Kashiwadacho, Ushiku, Ibaraki 300-1211 Japan; 3 Department of Medical Sciences, Graduate School of Comprehensive Human Sciences, University of Tsukuba, Tsukuba, 305-8575 Japan; 4 Department of Primary Care and Medical Education, Institute of Medicine, University of Tsukuba, Tsukuba, 305-8575 Japan

**Keywords:** Adverse drug events, Health literacy, Label comprehension, Medication package inserts

## Abstract

**Background:**

Self-medication using over-the-counter (OTC) medicines is one of the effective self-care measures in dealing with daily health problems. Health literacy (HL) is critical to ensuring the appropriate use of OTC medicines. The purpose of this study was to evaluate the association between HL and comprehension of medication package inserts among adults who use OTC medicines.

**Methods:**

We conducted a cross-sectional study using a self-administered questionnaire and interviews at 14 drugstores in the Kanto region in Japan from January to February 2020. The study participants were adults aged 20 years or older who purchased OTC medicines. HL was measured using the 14-item HL scale for Japanese adults (Japanese version of HLS-14), and comprehension of medication package inserts was evaluated using an interview survey (label comprehension study [LCS] form). The association between HL and LCS correct response rate and that between HL and attitude toward reporting adverse drug events (ADEs) were assessed using multiple linear regression and logistic regression analyses, respectively.

**Results:**

The analysis included the data of 140 adults, 50 men (35.7%) and 90 women (64.3%), with an average age of 55.2 years. The average HLS-14 score was 51.6, and the overall correct answer rate for reading comprehension was 57.5%. Multiple linear regression analysis revealed that a higher HLS-14 score was associated a higher LCS correct response rate (β = 1.01, *p* = 0.001). In addition, logistic regression analysis revealed that higher HL was associated with positive attitude towards reporting ADEs to health professionals (adjusted odds ratio = 1.06, *p* = 0.031).

**Conclusions:**

Adults with higher HL had higher comprehension of OTC package inserts, and higher HL was associated with positive attitude toward reporting ADEs to healthcare professionals. These results indicate that optimal self-medication with OTC medicines requires improving HL among the general public through health education and effective health information provision from pharmacists and registered sales clerks at drug stores.

**Supplementary Information:**

The online version contains supplementary material available at 10.1186/s40545-023-00596-3.

## Background

With the establishment of the self-medication tax system in 2017 for the special exemption of medical costs to promote the switching of drugs from prescription to over-the-counter (OTC) medicines, self-medication is being promoted in Japan as a means of reducing the frequency of hospital visits and the national healthcare burden [1]. The World Health Organization (WHO) defines self-medication as “the selection and use of medicines (including herbal and traditional products) by individuals to treat self-recognized illnesses or symptoms” [2]. The promotion of self-medication is expected to reduce unnecessary medical consultations and, eventually, health expenditure [3]. In contrast, there are risks and hazards associated with inappropriate self-medication, such as the interactions of OTC medicines with prescription drugs and adverse effects of OTC medicines [4]. The key to ensuring the safety of consumers practicing self-medication is the promotion of the rational use of medicines.

The WHO requires that adequate information is provided with medications to ensure the proper use of OTC medicines [5]. In Japan, the package of OTC drugs are labeled with medical information, and a document containing drug information is inserted in the packaged drug [6]. According to a previous survey, one-fourth of OTC customers answered that they did not read medication package inserts [7], and failure to check medication labels or package inserts can lead to the misuse of OTC medicines and adverse drug events (ADEs) [8].

In recent years, health literacy (HL) has often been cited as an indicator of a patient’s knowledge about health. According to a systematic review on HL, “HL is linked to literacy and entails people’s knowledge, motivation, and competences to access, understand, appraise, and apply health information in order to make judgments and take decisions in everyday life concerning healthcare, disease prevention, and health promotion to maintain or improve quality of life during the life course” [9]. Optimal medication use requires general HL skills such as appropriate understanding of one’s own illness, follow-up on instructions outlined on medication labels, and adherence to instructions provided by pharmacists [10]. Therefore, it is assumed that individuals with high HL can generally practice proper self-medication, whereas those with low HL tend to misinterpret label instructions and use medicines inappropriately, with the potential risks of serious consequences. Although the importance of HL has been studied and identified to be critical for the management of the adverse effects of alternative medicines in patients with chronic disorders [11], no study in Japan has comprehensively evaluated the HL of OTC medicine consumers, their comprehension of OTC medication labels or package inserts, and their attitude toward reporting ADEs.

This study aimed to describe the general public on their HL in the use of OTC medicines in community drugstore settings and to evaluate the association between HL and comprehension of medication package inserts and between HL and attitude toward reporting ADEs to healthcare professionals. Based on our findings, we discussed potential approaches for promoting the optimal use of OTC medicines by the general public.

## Methods

### Study population

The present survey was conducted as a cross-sectional study. The study participants were 151 adults aged 20 years or older who purchased OTC medicines on the day of investigation at 14 randomly selected stores belonging to two drugstore chains (Welcia Yakkyoku Co., Ltd. and HAC Drug) in the Kanto region, Japan.

The drugstore sites were selected using the two-stage cluster sampling. Cities with more than and less than 200,000 inhabitants (the criteria for a major urban city in Japan) were stratified into urban and non-urban areas, respectively, and sample stores were randomly selected from within each cluster. The participants were considered eligible for the survey if they were aged 20 years or older and purchased OTC medicines on the day of the surveys. OTC medicines are categorized as follows: nonprescription medicines with mandatory face-to-face instruction from a pharmacist (guidance-mandatory drugs); general sale with instruction from a pharmacist (General Sale Risk Class: GSC 1); general sale, with instruction recommended (GSC 2); and general sale, with instruction provided upon request (GSC 3) [12]. In this study, all customers who bought OTC medicines in these categories were included. They were considered ineligible if they did not provide consent for participation, were unable to answer the questionnaires independently.

### Data collection

The survey was conducted at each drugstore in one day from January to February 2020. A request for participation was orally provided to candidate adults who had purchased OTC medicines (i.e., guidance-mandatory drugs and GSC 1, 2, and 3 drugs) at the store. Subsequently, those who agreed to participate in the research answered a self-administered questionnaire and label-comprehension interview questionnaire. The participants were recruited using sequential sampling at each research site (drugstore employees or investigators spoke to candidate participants and asked them to answer questions from the self-administered and interview questionnaires).

The first part of the survey, a self-administered questionnaire, collected information on participants’ personal characteristics including age, gender, educational attainment, household income, and consultation with pharmacists or registered sales clerks at drug stores on purchase of OTC medicines. This part of the survey also included an HL assessment using the 14-item HL scale for Japanese adults (Japanese version of HLS-14) [13], and a question about behaviors or actions in response to ADEs (i.e., attitude toward asking or reporting events to pharmacists). The second part of the survey, an interview questionnaire, evaluated participants’ comprehension of medication package inserts (Japanese version of the label comprehension study [LCS] form) [14].

### Explanatory variable: health literacy

In this study, we used the Japanese version of HLS-14 [13], which has been assessed for validity and reliability and verified for use among the general public in Japan. HLS-14 is composed of 14 items related to HL, and each is scored on a 5-point Likert scale, with total scores ranging from 14 to 70; a higher score means higher health literacy. The questionnaire was used in the current survey with permission from the original developers. The total HLS-14 score consists of 3 domains including functional, communicative, and critical HL. Explanation of each domain is as follows. Functional literacy: basic skills in reading and writing to function effectively in everyday situations. Communicative literacy: more advanced skills to participate actively in everyday activities, to extract information and derive meaning from different forms of communication, and to apply new information to changing circumstances. Critical literacy: more advanced skills to critically analyze information and use this information to exert greater control over life events and situations [15].

### Outcome variable: label comprehension study and attitude toward reporting adverse drug events to healthcare professionals

Comprehension of OTC medication labels was evaluated using the method described by Hashiguchi [14]. The LCS questionnaire was used with permission from the original developers. It consists of 14 items related to sample medication label information, and participants were requested to complete the LCS questionnaire after reading the package insert of the representative OTC medicine, “famotidine”. The questionnaire required the participants to report their reaction to observed health problems while taking the medicine and the reason for their answer. The LCS form does not only assess one’s knowledge about medicines but their actions toward a given situation related to medicine intake, which assesses a higher dimension of HL. The original questionnaire contained two questions, i.e., Q3 and Q5, which we thought may be difficult for consumers to answer; therefore, we partially modified the questionnaire after repeated discussions among the researchers, although the modified questionnaire was not strictly validated. Face-to-face interviews were conducted by physicians or pharmacists. The correct response rate was calculated for the aggregate and individual question items in a manner similar to that performed in a previous study [14]. In addition, the correct response rate for individual participants was calculated to assess the association between the level of HL and comprehension of medication labels. Participants’ answers were judged to be correct if both the response and reason were correct.

Attitude toward reporting ADEs to healthcare professionals was assessed using the question, “Do you contact and consult a doctor or pharmacist if adverse drug events occur?” The answer was dichotomized as “Yes” and “No”, which corresponded to positive and negative attitudes towards reporting ADEs, respectively.

### Statistical analysis

First, we performed descriptive analysis of the HL scores and the correct response rate of the LCS questions. Second, the association between HL and the LCS correct response rate was analyzed using univariate and multivariate analyses. For the univariate analysis, Spearman’s rank correlation coefficient was calculated. Multiple regression analysis was performed for the multivariate analysis. Third, the association between HL and attitude toward reporting ADEs to healthcare professionals was assessed. Univariate analysis including Student’s *t*-test or Chi-square test was performed to assess the association between each independent variable and the outcome. Logistic regression analysis was also performed to assess the association between HL and the outcome. In each multivariate analysis, age, gender, and variables with a *p*-value < 0.10 in the univariate analysis were included as covariates. Participants with missing data, including age, gender, or HLS-14 scores, were excluded from the analyses by pairwise deletion. After conducting the analysis using the total score of HL, exploratory analyses were conducted using the same multivariate models to assess the association between each subscale of HL and the outcome variables. The multiple regression analysis results are reported as β and standard errors (SE). Those of the logistic regression analysis are presented as odds ratios (OR) and 95% confidence intervals (CI). Using G power analysis, we estimated a sufficient sample size of 55 with an α set at 0.05 and power set at 0.8, assuming a medium effect size and 5 independent variables. All analyses were performed using SPSS version 26 (IBM, Armonk, NY, USA), and statistical significance was set at *p* < 0.05 (two-sided).

### Ethical considerations

The survey was administered to those whom the researcher judged to have no significant physical or mental burden in responding to the survey. The participants answered the question after the researchers had explained that the participant could decide not to answer a question if they did not want to. Furthermore, we informed the participants that they could stop answering the questions at any time during data collection. Ethical approval was obtained from the Ethical Committee of the University of Tsukuba, Faculty of Medicine, Japan (Approval No. 1477).

## Results

### Characteristics of participants

A total of 151 adults participated in this study. After deleting 11 cases with missing data on age, gender, or HL, the data of 140 participants were analyzed (men, 50 [35.7%] and women, 90 [64.3%]), with an average age of 55.2 years (± SD 15.3). The basic characteristics are shown in Table 1. The OTC medicines that were mostly purchased were cold remedies, antipyretic analgesics, and stomach medicines, and the majority were GSC 2 medications (see Additional file 1). Only 12.9% of the consumers indicated that they consulted pharmacists or registered sales clerks about drug information at the time of purchasing the OTC medicines.

**Table 1:** Basic characteristics of the study population (*n* = 140)

Characteristics	
Age, mean (SD)	55.2 (15.3)
Gender, *n* (%)	
Women	90 (64.3)
Men	50 (35.7)
Educational attainment, *n* (%)	
Junior or senior high school	65 (46.4)
Vocational college, technical school, or junior college	43 (30.7)
University undergraduate or graduate	29 (20.7)
Others or missing	3 (2.1)
Household income (yearly income), *n* (%)	
< 3 million yen	24 (17.1)
3–5 million yen	40 (28.6)
≥ 5 million yen	45 (32.1)
Unanswered^a^	28 (20.0)
Missing	3 (2.1)
Consultation with pharmacists or registered sales clerks at drugstores on purchase of OTC medicines, *n* (%)	
Yes	18 (12.9)
No	121 (86.4)
Do not remember or unanswered	1 (0.7)

*SD* standard deviation, *OTC* over-the-counter

^a^The unanswered category meant a voluntary refusal to answer a question

### Health literacy and label comprehension study

HL analysis revealed that the average functional, communicative, and critical subscales scores and the total HL score was 18.9 (3.8), 18.0 (4.7), 14.7 (3.3), and 51.6 (7.4), respectively (Table 2). Cronbach's alpha (0.77, 0.88, 0.81, and 0.76, respectively) confirmed that the internal consistency of the questionnaires was satisfactory. The average label comprehension rate among participants was 57.5% (see Additional file 2).

**Table 2:** Summary of the total health literacy and subscales scores (*n* = 140)

Subscale	Functional	Communicative	Critical	Total
Mean score (SD)	18.9 (3.8)	18.0 (4.7)	14.7 (3.3)	51.6 (7.4)
Cronbach’s alpha	0.77	0.88	0.81	0.76

*SD* standard deviation

### Association between health literacy and comprehension of medication package inserts

The correlation between total HLS-14 score and level of comprehension of medication package inserts was evaluated using Spearman’s rank sum test. The correlation coefficient was low (correlation coefficient [*r*] = 0.395, *p* < 0.001) but statistically significant. Evaluation of the association between the HL subscales scores and comprehension of medication package inserts showed a significant correlation of package inserts comprehension with communicative and critical HL (*r* = 0.304, *p* = 0.002; *r* = 0.358, *p* < 0.001, respectively) and no correlation with functional HL (*r* = 0.087, *p* = 0.40).

Multiple regression analysis adjusting for age, gender, educational attainment, and annual income revealed that higher HL was associated with higher LCS scores (β = 1.01, *p* = 0.001; Table 3). In a separate model with the subscales of HL, higher communicative and critical HL were significantly associated with higher LCS scores (β = 1.46, *p* = 0.003 and β = 2.47, *p* = 0.001, respectively), but functional HL was not (β = 0.14, *p* = 0.81).

**Table 3:** Factors associated with the label comprehension study correct response rate (*n* = 94)

Variable	β	SE	*p* value
Age	− 0.652	0.158	< 0.001
Gender, women	2.72	5.00	0.59
Educational attainment			
Ref.: Junior or senior high school			
Vocational college, technical school, or junior college	2.38	5.50	0.67
University undergraduate or graduate	7.54	6.13	0.22
Annual household income			
Ref: < 3 million yen			
3–5 million yen	− 1.39	7.73	0.86
≥ 5 million yen	12.06	7.32	0.10
Unanswered	1.02	8.01	0.90
HLS-14 total score	1.01	0.30	0.001

*SE* standard error, *HLS-14* the 14-item health literacy scale

### Association between health literacy and behaviors related to addressing adverse drug events

To the question “Do you contact and consult a doctor or pharmacists if adverse drug events occur?” 97 participants (69.3%) answered “Yes” and 40 (28.6%) answered “No”. The average total HLS-14 score of those who answered “Yes” and “No” was 52.8 and 48.7 (*p* = 0.003), respectively, indicating a significant association between participants who indicated that they would ask a doctor or pharmacist for guidance and higher HL scores. Analysis of subscales showed a significant association between higher communicative and critical HL scores and positive behavior towards seeking guidance for adverse reactions (average communicative and critical HL score for the positive vs negative behavior toward reporting ADEs; 18.7 vs 16.3, *p* = 0.007 and 15.3 vs 13.5, *p* = 0.003, respectively). In contrast, no significant relationship was found with functional HL score (average functional HL score for the positive vs negative behavior toward reporting ADEs; 18.8 vs 19.0, *p* = 0.86). Logistic regression analysis adjusted for by age, gender, and educational attainment revealed that higher HL was associated with the intention to report ADEs to health professionals (adjusted OR = 1.06, 95% CI 1.01–1.12, *p* = 0.031; Table 4). In a separate model with the HL subscales, higher communicative HL was significantly associated with the intention to report ADEs (adjusted OR = 1.08, 95% CI 1.00–1.17, *p* = 0.049), but functional and critical HL were not (adjusted OR = 1.00, 95% CI 0.90–1.11, *p* = 0.999; adjusted OR = 1.13, 95% CI 1.00–1.28, *p* = 0.052, respectively).

**Table 4:** Association between health literacy and positive attitude toward reporting adverse events (*n* = 137)

	Adjusted OR (95% CI)*	*p* value
HLS-14 total score	1.06 (1.01–1.12)	0.031
Domain scores		
Functional health literacy score	1.00 (0.90–1.11)	0.999
Communicative health literacy score	1.08 (1.00–1.17)	0.049
Critical health literacy score	1.13 (1.00–1.28)	0.052

*95% CI* 95% confidence interval

These results were obtained by logistic regression analysis

All models were adjusted for age, gender, and educational attainment

Each score was included separately in the model

## Discussion

The present study showed the level of HL competency among people who purchased OTC medicines at community drugstores. We also demonstrated that higher HL was associated with higher comprehension of medication package inserts and was associated with a higher rate of expected consultation with healthcare professionals on ADEs.

The result indicated that HL was associated with the comprehension of medication package inserts: individuals with higher HL could better understand information presented in medication package inserts. This result is consistent with the findings from previous studies that demonstrated that poor HL is associated with lower levels of comprehension of label information [16–18]. Further, inadequate understanding of drug labels can lead to improper self-medication and poor adherence to medications [19–21]. In addition, the present study confirmed a significant association between two subscales, communicative and critical HL, and level of label comprehension. This finding suggests that the level of comprehension of medication package inserts is associated with consumers’ ability to obtain additional information from people around them and to critically appraise information contained in medication package inserts. Therefore, health education focusing on communicative or critical HL, rather than just providing knowledge, would be effective in improving the understanding of medication package inserts. Furthermore, development of more readable and an improved format could contribute to the understanding of medication package inserts [22].

A previous study conducted in Japan measured HL in patients who were taking complementary and alternative medicines and revealed that HL was higher among those who reported ADEs to their attending physician than among those who did not [11]. Consistent with that study, the current study identified an association between higher total HL score and positive attitude toward reporting ADEs to a doctor or pharmacist. Similar to the results of the subscale analysis by Yukawa et al. [11], the subscale analysis in the present study demonstrated significant associations between communicative HL and positive attitude toward reporting adverse reactions to healthcare professionals. The ability for consumers to exchange or share information could lead to positive behaviors toward reporting ADEs.

Despite the need to obtain guidance from healthcare professionals for the proper use of OTC medicines, only a small number of consumers indicated that they consulted pharmacists or registered sales clerks about drug information when purchasing OTC medicines. In addition, the results of our analyses indicate the need to implement measures to educate OTC users on the importance of contacting and consulting pharmacist at drugstores when they experience ADEs. Overall, the findings of this study suggest the need for more proactive interventions by pharmacists or registered sales clerks in drugstores to complement communicative HL and ensure optimal self-medication.

There were several limitations in this study. First, there may have been selection bias because the participants were consumers who purchased OTC medicines at drugstores. However, the total HL and subscales scores were similar to those reported in a previous study [13]. Second, the validity of findings related to the comprehension of medication package inserts is unclear. It was difficult to complete the LCS of consumers at the drugstores because the interviews were conducted under limited time constraints and participants’ tendency to respond depended on their individual medical knowledge. Nevertheless, participants’ comprehension determined in this study was similar to that of participants in a previous study [14]. Future research that measures the comprehension of OTC medication package inserts should be conducted to develop more convenient and scientifically valid tools or indicators. Third, the LCS responses had several missing data, and the number of analyzed data from the LCS responses was decreased. This may be due to the time-consuming nature and complexity of answering the LCS questions. Although this can lead to bias, the relationship between HL and the LCS correct response rate might be more robust if the missing data were less.

## Conclusions

We identified significant associations between higher HL scores and higher levels of comprehension of medication package inserts information and positive attitude toward reporting ADEs to healthcare professionals. To promote appropriate self-medication, measures to support OTC medicine users, such as encouraging the provision of health information or promoting communication with distributors of OTC medicines (i.e., pharmacists and registered sales clerks at pharmacies), according to consumers’ HL level are warranted.

## Supplementary Information

**Additional file 1**: Class and type of OTC medicines purchased at pharmacies (n = 140). This file outlines the classes of OTC medicine and the specific OTC medicines purchased by the participants from the drugstores on the day of data collection with their respective frequencies.

**Additional file 2**: Results of the label comprehension study using famotidine medication labels. This file outlines the number of participants who answered correctly to questions on participants’ reactions toward occurrences within the duration of medication consumption.

## Data Availability

The datasets used and analyzed during the current study are available from the corresponding author on reasonable request.
