# Supplementary material for: Association between health literacy and medication comprehension; attitudes toward reporting adverse events in adults using over-the-counter medicines
Source: J Pharm Policy Pract. 2023 Jul 17;16:90. doi: 10.1186/s40545-023-00596-3 (PMC10351140; doi:10.1186/s40545-023-00596-3)
Supplement: Supplementary file 1 — Additional file 1: Class and type of OTC medicines purchased at pharmacies (n = 140). This file outlines the classes of OTC medicine and the specific OTC medicines purchased by the participants from the drugstores on the day of data collection with their respective frequencies. [file 40545_2023_596_MOESM1_ESM.docx]

Additional file 1. Class and type of OTC medicines purchased at pharmacies (n = 140)

| Characteristic | n (%) |
| --- | --- |
| **Class** |  |
| Guidance-mandatory and GSC1 | 5 (3.6) |
| GSC 2 | 106 (75.7) |
| GSC 3 | 29 (20.7) |
| **Type of OTC medicine** |  |
| Cold remedy | 27 (19.3) |
| Eye drop | 10 (7.1) |
| Antipruritic | 4 (2.9) |
| Compress | 11 (7.9) |
| Stomach medicine | 14 (10.0) |
| Motion sickness medicine | 3 (2.1) |
| Antipyretic analgesic | 12 (8.6) |
| Cough medicine | 5 (3.6) |
| Disinfectant | 2 (1.4) |
| Vitamin | 9 (6.4) |
| Other or unknown | 43 (30.7) |

OTC, over-the-counter medicine; GSC, General Sale Risk Class

Guidance-mandatory drug: nonprescription medicines with mandatory face-to-face instructions from a pharmacist. GSC 1: drugs with adverse effects can damage a patient’s health to a degree that can affect their daily life; that are designated by the Minister of Health, Labour and Welfare as requiring special attention for use; and with active ingredients that are distinctly different from those of drugs that are approved for production and sales within a certain period of time. GSC 2: drugs with adverse effects that can damage a patient’s health to a degree that can affect their daily life (excluding GSC 1 drugs) and have been designated by the Minister of Health, Labour and Welfare as drugs that require special attention for use. GSC 3: OTC drugs other than GSC 1 and GSC 2 drugs (8).
