# Supplementary material for: Association between health literacy and medication comprehension; attitudes toward reporting adverse events in adults using over-the-counter medicines
Source: J Pharm Policy Pract. 2023 Jul 17;16:90. doi: 10.1186/s40545-023-00596-3 (PMC10351140; doi:10.1186/s40545-023-00596-3)
Supplement: Supplementary file 2 — Additional file 2: Results of the label comprehension study using famotidine medication labels. This file outlines the number of participants who answered correctly to questions on participants’ reactions toward occurrences within the duration of medication consumption. [file 40545_2023_596_MOESM2_ESM.docx]

Additional file 2. Results of the label comprehension study using the package insert of “famotidine”

|  | Items | Correct answer (%) |
| --- | --- | --- |
| Q1 | A case of sore throat and high fever (n = 106) | 42.5 |
| Q2 | A case of pregnancy (n = 111) | 80.2 |
| Q3 | Concomitant use of other stomach medicine (n = 107) | 54.2 |
| Q4 | A case of past allergic symptoms (n =110) | 49.1 |
| Q5 | A case of no problems with medication intake^†^ (n = 111) | 71.2 |
| Q6 | A case of worsening constipation symptoms (n = 110) | 37.3 |
| Q7 | Symptoms of serious side effects (n = 109) | 65.1 |
| Q8 | Actions in a case of overdose (n = 110) | 56.4 |
| Q9 | A case of leukopenia (n = 110) | 66.4 |
| Q10 | A case of an elderly patient (n = 110) | 47.3 |
| Q11 | Actions when a side effect occurs (n = 107) | 53.3 |
| Q12 | A case of a lactation mother (n = 109) | 75.2 |
| Q13 | Actions when symptoms remain after 2 weeks of medication consumption (n = 108) | 63.0 |
| Q14 | A case of bronchial asthma (n = 110) | 40.9 |
| Average (n = 97) | | 57.5 |

^†^Questions 3 and 5 were modified from the original questionnaire.

Q3: Concomitant use with laxative medicine→Concomitant use of other stomach medicine

Q5: A case of history of childhood asthma with no medication→A case without any health problems
